# Slaughter analysis, incidence of myopathy and breast muscle characteristics of broiler chickens fed crude fibre concentrate feeds

**DOI:** 10.2478/jvetres-2025-0033

**Published:** 2025-06-11

**Authors:** Jakub Urban, Damian Bień, Arkadiusz Matuszewski, Patrycja Ciborowska, Anna Zalewska, Dorota Pietrzak, Marta Chmiel, Adriana Jaroszek, Lucas Elzie Graham, Monika Michalczuk

**Affiliations:** 1 Department of Animal Breeding and Nutrition, 02-786 Warsaw, Poland; 2 Department of Animal Environment Biology, Institute of Animal Sciences, Warsaw University of Life Sciences, 02-786 Warsaw, Poland; 3 Department of Food Technology and Assessment, Institute of Food Sciences, Warsaw University of Life Sciences, 02-787 Warsaw, Poland; 4 ADDICOO GROUP s.r.o., 787 01 Šumperk, Czech Republic; 5 J. Rettenmaier USA, Schoolcraft, MI 49087, USA

**Keywords:** breast muscle quality, chicken broiler, crude fibre, slaughter performance

## Abstract

**Introduction:**

This study investigated the impact of adding crude fibre concentrate (CFC) to broiler chicken diets on slaughter results and breast muscle quality.

**Material and Methods:**

A total of 990 male Ross 308 chicks were divided into control (C), experimental 1 (A1) and experimental 2 (A2) groups. Experimental diets contained CFC at different levels: A1 had 0.4% in the starter diet, 0.8% in the first grower diet, 0.8% in the second grower diet and 0.2% in the finisher diet, and A2 had 0.6%, 1.0%, 1.2% and 0.4% in the same diets. On day 42, 20 birds per group were slaughtered and dissected. Breast muscles were weighed and visually assessed for myopathic defects (white striping, wooden breast and “spaghetti” meat). The tissue was also analysed for residual myopathy incidence and associated physicochemical properties, namely drip loss, shear force, pH, water holding capacity, collagen content, colour parameters and basal chemical composition.

**Results:**

Consumption of the CFC additive statistically significantly reduced (P-value ≤ 0.05) white striping defects and increased final live weight by 2.1% for birds in group A1 and by 3.3% in group A2. Group A1 carcasses also weighed 6.7% more and group A2 carcasses 4.1% more. Additionally, A1 carcasses yielded 1.5% more and A2 carcasses 0.8% more leg muscle, also statistically significantly greater yields than C carcasses (P-value ≤ 0.05). The slaughter yield of birds in group A1 was 3.3% higher (P-value ≤ 0.001) than that of birds in group C. The use of the CFC additive in the diets of both experimental groups had no adverse effect on the other analysed parameters.

**Conclusion:**

Crude fibre concentrate addition to the complete feed mixture is recommended for improving the results of the slaughter analysis and the visual quality of meat.

## Introduction

Nutrition is one of the most important factors affecting poultry production. Over the last 30 years, the production of broiler chickens worldwide has increased significantly (26). As production has increased, consumer pressure to ban antibiotic growth promoters (AGPs) has also increased. The introduction of legislation prohibiting antibiotics in feed used by the poultry industry has led to an increased incidence of intestinal disorders in broilers. This turn of events has presented the poultry industry and research community with the crucial task of finding alternatives that will improve the health and function of the gastrointestinal tract (17, 20). One of the more effective alternatives to AGPs may be fibre. Unfortunately, results from earlier studies have shown an adverse effect of fibre on the digestibility of the nutrients in forage (17, 20) and on growth performance (20, 41). The importance of fibre as one of the primary feed nutrients has been almost completely ignored due to its low nutritional value (from a chemical point of view). However, after a new series of experiments, it has been shown that the unique physicochemical characteristics of insoluble crude fibre fractions can benefit the development and function of the digestive tract. Consequently, the addition of fibre can lead to improved health and increased productivity in broiler chickens (13, 19, 27, 33, 36). According to Mateos *et al*. (28), to enhance the positive effects of supplemental crude fibre on productive performance and physiological processes in the bird’s body, the fibre should undergo a lignification process. The resulting lignocellulose comprises carbohydrates (cellulose, hemicellulose) and aromatic polymers (lignin). The physical properties and exact composition of the various lignocellulose fractions depend on the source from which the fibre was obtained (16, 20). Lignocellulose positively affects faecal consistency by making faeces and consequently litter drier, and also improves intestinal microbiota, fermentation activity and protein digestibility in broiler chickens (5, 7, 20, 32, 44). This study aimed to determine the effect of adding CFC to broiler chickens’ diet on the slaughter results, the incidence and extent of myopathy and the physicochemical properties of breast muscle.

## Material and Methods

### Crude fibre concentrate

The crude fibre concentrate used (ARBOCEL; J. Rettenmaier & Söhne, Rosenberg, Germany) is distinguished by a water content of 7.7% and an extremely high water-binding capacity (WHC) of up to 800%. It contains 65.3% crude fibre, 25.1% non-protein nitrogen compounds, 1.0% total protein, 0.3% crude fat and 0.5% crude ash (6, 34, 44, 45). ARBOCEL crude fibre concentrate is a product that consists of lignocellulose derived from debarked and thoroughly cleaned spruce (*Picea*) trees. It is mycotoxin-free. This CFC stimulates the work of intestinal villi and enhances the enzymatic activity of the gastrointestinal tract).

### Animals

The study utilised Ross 308 male chicks (cockerels). A total of 990 chicks, all one-day-old, were purchased and divided into three equal groups according to Urban *et al*. (44), these being a control (C) and two experimental groups (A1 and A2). The experimental groups differed in the proportion of the diet which was CFC. Rearing lasted 42 days in standard conditions and was to a stocking density of less than 33 kg/m^2^ on the final day. The chickens were housed on a floor system with wood pellets, with a light cycle that was maintained according to the Ross Management Guide (37), and had *ad libitum* access to fresh water.

Throughout the rearing period, the broilers were fed a starter diet on days 1–10, a grower diet on days 11–21, an adjusted grower diet on days 22–35 and a finisher diet on days 36–42. Group A1 had 0.4% CFC in the starter diet, 0.8% in the first grower diet, 0.8% in the second grower diet and 0.2% in the finisher diet and A2 had 0.6%, 1.0%, 1.2% and 0.4% in the same diets. The composition of the feed mixtures used and the averaged results of the NIR (near-infrared) analysis of the feed samples were collated and published in a manuscript on the same experiment but focusing on welfare parameters and the caecal microbiome (44).

### Selection of animals and post-slaughter handling

From each group, 20 chicks with a body weight close to the average of the group were selected for slaughter. The slaughter of chickens and the post-slaughter treatment of carcasses were carried out using the industrial method following the technical and sanitary requirements in force in the poultry industry. After that, the carcasses were cooled at 4°C for 24 h. From the storage stage, the following elements were extracted and weighed from each chilled carcass: the liver, gizzard, heart and abdominal fat. Then, each carcass was weighed and dissected to obtain the pectoral muscles and leg muscles, which were also weighed. All the extracted weights were used for subsequent calculations of slaughter yield and the percentage of the final live weight which was selected organs and muscles.

### Assessment of pectoral muscles for qualitative and visual signs of myopathies

Appropriate assessment scales were used to assess the presence and intensity of visual myopathies of the pectoral muscle: white striping (WS) according to the method proposed by Kuttappan *et al*. (23), spaghetti meat (SM) according to the scale proposed by Baldi *et al*. (3) and wooden breast (WB) according to the method proposed by Khalil *et al*. (18) with scores given in the range of 0 (normal muscle) to 3 (markedly defective).

### Determination of drip loss

To determine drip loss, each left breast muscle was weighed, dried (using a paper towel), and weighed again after 24 h of cold storage (4°C). The weight difference obtained after the calculation (weight of left breast muscle before cooling – dried weight of this muscle after 24 h at 4°C = drip loss) determines the water loss.

### Determination of shear force

The shear force of each pectoral muscle was determined according to the method described by Michalczuk *et al*. (29).

### Determination of pH measured 24 h post slaughter, WHC, basic chemical composition and collagen content of breast muscles

Breast muscle samples were prepared for further analyses and pH measurements were taken in the same way that they were by Michalczuk *et al*. (29). Drip loss and WHC were analysed with the protocol published by Michalczuk *et al*. (31), and the elemental chemical composition of meat and collagen content was determined using a near-infrared (NIR) method also developed by Michalczuk *et al*. (30).

### Determination of colour parameters (L* (lightness), a* (redness) and b* (yellowness))

Colour measurement of the examined breast muscles was performed using a CR-410 trichromatic spectrophotometer from Konica Minolta (Tokyo, Japan). The absolute colour difference ΔE (between the colour of breast muscles obtained from chickens in the C group and its colour from chickens in the A1/A2 groups) was calculated using the following equation (1):

$$\Delta E=\sqrt{{\left(L_{1}^{*}-L_{2}^{*}\right)}^{2}+{\left(a_{1}^{*}-a_{2}^{*}\right)}^{2}+{\left(b_{1}^{*}-b_{2}^{*}\right)}^{2},}$$

where ΔE is absolute colour difference, L*_1_, a*_1_ and b*_1_ are colour parameters of the breast muscles obtained from chickens from the control group (C) and L*_2_, a*_2_ and b*_2_ are colour parameters of the breast muscles obtained from chickens from the experimental groups (A1 or A2). The obtained ΔE values were interpreted according to Bendowski *et al*. (4).

### Statistical analysis

The results obtained were statistically analysed as per Urban *et al*. (44).

## Results

### Slaughter analysis and organ weights

Birds in group A2 were determined to have the average highest final live weight (Table 1). However, this did not coincide with the highest average carcass weight or fertility yield results, which came from group A1 birds. The proportion of leg muscle tissue in body weight was also highest in group A1. The percentage of live weight which was liver in group A2 was 2.41 and was higher (P-value ≤ 0.05) than the percentage of liver in groups C and A1. Based on the analysis of the other results presented in Table 4, it could be concluded that there was no adverse effect of adding crude fibre concentrate on the percentages of live weight which were the other elements obtained from chicken carcasses. The breast muscles, hearts, gizzards and abdominal fat were those other elements and accounted for similar live weight percentages across all groups.

**Table 1.** Production results, carcass yields and body component percentages of selected organs and muscles of broilers fed a normal diet and fed a diet containing crude fibre concentrate

Parameter	Group			SEM	P-value
	C	A1	A2		
Final live weight (g)	2,986.20^a^	3,049.20^ab^	3,084.60^b^	16.945	0.049
Carcass weight (g)	2,148.30^A^	2,291.90^B^	2,236.45^B^	24.054	0.003
Slaughter yield (%)	71.92^A^	75.18^B^	72.50^A^	1.490	≤0.001
^1^Body components					
Liver (%)	2.04^A^	2.01^A^	2.41^B^	0.058	0.004
Gizzard (%)	0.59	0.62	0.56	0.015	0.310
Heart (%)	0.49	0.43	0.48	0.014	0.113
Abdominal fat (%)	0.71	0.82	0.78	0.054	0.697
Breast muscles (%)	21.98	22.82	22.14	0.259	0.380
Leg muscles (%)	13.35^A^	14.85^B^	14.17^AB^	0.199	0.005

A, B – P-value ≤ 0.01;

a, b – P-value ≤ 0.05;

1 C – control group; A1 – group fed crude fibre concentrate in 0.2–0.8% proportions across four development-stage diets; A2 – group fed crude fibre concentrate in 0.4–1.2% proportions across the four diets; SEM – standard error of the mean.

1 – Body component percentages were calculated on the final live weight

**Table 2.** Visual assessment of the intensity of myopathy in the pectoral muscles of broilers fed a normal diet and fed a diet containing crude fibre concentrate

Indicator	Statistic	Score	Group					
			C		A1		A2	
			%	n	%	n	%	n
White striping		0	31.3	5	75.0	12	75.0	12
		1	37.5	6	25.0	4	18.8	3
		2	31.3	5	0.0	0	6.3	1
		3	0.0	0	0.0	0	0.0	0
Kruskal–Wallis test	P-value = 0.006		A		B		B	
Spaghetti meat (SM)		0	100.0	16	100.0	16	100.0	16
		1	0.0	0	0.0	0	0.0	0
		2	0.0	0	0.0	0	0.0	0
Kruskal–Wallis test	-							
Wooden breast		0	100.0	16	100.0	16	100.0	16
		1	0.0	0	0.0	0	0.0	0
		2	0.0	0	0.0	0	0.0	0
		3	0.0	0	0.0	0	0.0	0
Kruskal–Wallis test	-							

1 ^A, B^ – P-value ≤ 0.01; C – control group; A1 – group fed crude fibre concentrate in 0.2–0.8% proportions across four development-stage diets; A2 – group fed crude fibre concentrate in 0.4–1.2% proportions across the four diets

### Visual and tactile assessment of pectoral muscles for the presence of quality defects

Visual and tactile tests showed that the lowest (P-value ≤ 0.01) number of breast muscles with a WS defect was recorded for tissue obtained from chickens of the A1 and A2 groups, and the highest (P-value ≤ 0.01) from the C group. The WB and SM breast muscle defects were not found in meat obtained from carcasses from either the C or A1 and A2 groups.

### Drip loss, shear force, pH and WHC of breast muscles

The average pH, drip loss, WHC and shear force results are presented in Table 3. No significant differences in these meat qualities were found between the control group and the experimental groups.

**Table 3.** Physicochemical quality of breast muscles of broilers fed a normal diet and fed a diet containing crude fibre concentrate

Quality characteristic	Group			SEM	P-value
	C	A1	A2		
Drip loss (%)	3.10	2.90	2.40	0.187	0.298
Shear force (N)	53.93	56.30	46.92	1.967	0.128
pH	5.94	5.94	5.95	0.015	0.971
WHC (cm^2^/g)	3.94	3.72	3.76	1.026	0.886

1 C – control group; A1 – group fed crude fibre concentrate in 0.2–0.8% proportions across four development-stage diets; A2 – group fed crude fibre concentrate in 0.4–1.2% proportions across the four diets; SEM – standard error of the mean

### Basic chemical composition of breast muscles

Table 4 shows the average content of water, protein and fat in the broiler chicken breast muscles. The results indicated no significant differences between group C and groups A1 and A2. The addition of crude fibre concentrate to the feeds for chickens from groups A1 and A2 did not change the water, protein, fat or collagen content of the breast muscles.

**Table 4.** Basic chemical composition of breast muscles of broilers fed a normal diet and fed a diet containing crude fibre concentrate

Content (%)	Group			SEM	P-value
	C	A1	A2		
Water	74.98	74.29	74.52	0.107	0.053
Protein	22.62	22.55	22.70	0.104	0.858
Fat	2.23	2.44	2.27	0.089	0.595
Collagen	1.08	1.09	0.96	0.029	0.139

1 C – control group; A1 – group fed crude fibre concentrate in 0.2–0.8% proportions across four development-stage diets; A2 – group fed crude fibre concentrate in 0.4–1.2% proportions across the four diets; SEM – standard error of the mean

### L*, a*, b* colour parameters

In Table 5, the average results for the L*, a* and b* colour parameters are shown. There were no significant differences in the analysed colour parameters between the control group and experimental groups. The ΔE values for the experimental groups did not exceed 1.0, *i.e*. the difference in colour between the breast muscles obtained from control group chickens and those obtained from experimental group birds was visually undetectable. The addition of crude fibre concentrate to the feeds for chickens in groups A1 and A2 did not affect the colour of breast muscles.

**Table 5.** Colour parameters of breast muscles of broilers fed a normal diet and fed a diet containing crude fibre concentrate

Colour parameter	Group			SEM	P-value
	C	A1	A2		
L* (lightness)	53.73	53.37	52.88	0.366	0.650
a* (redness)	10.98	10.44	11.27	0.253	0.407
b* (yellowness)	16.08	16.20	15.69	0.168	0.488
ΔE	0.00	0.66	0.98	-	-

1 C – control group; A1 – group fed crude fibre concentrate in 0.2–0.8% proportions across four development-stage diets; A2 – group fed crude fibre concentrate in 0.4–1.2% proportions across the four diets; SEM - standard error of the mean

## Discussion

The group of factors that significantly determine the effect of fibre supplementation in feed on the production parameters of broiler chickens can include the source of the fibre (*i.e*. soluble *vs* insoluble), the particle size, the contents of energy and protein (*i.e*. amino acids) in the feed mixture used, the extent and duration of the supplementation’s incorporation into the rearing procedure, and the age and physiological status of animal (1, 12, 15, 40–43). However, the first factor is the major one, since most studies report changes in production yield due to the addition of insoluble fibres to the feed mixture (43), which are the basic structural elements of crude fibre. Insoluble fibre fractions mainly contain the insoluble parts of the plant cell wall, *i.e*. three-dimensionally arranged fibrous polysaccharides such as cellulose, hemicellulose and/or encrusting non-saccharide substances such as lignin (11, 43). Cellulose, hemicellulose and lignin are the main components that make up the lignocellulose complex, which has been the subject of research in the last decades focusing on the use of an innovative source of insoluble dietary fibre (36, 49). Chicken broilers fed with a compound feed of which 0.6% was lignocellulose (replacing some soybean meal and corn) were characterised by a higher final live weight than those in the control group or the group provided a feed with 0.4% lignocellulose in the bill (25, 36). The use of 0.8% lignocellulose in feed mixture for broiler chickens significantly improved carcass yield (7, 25), and 0.75% lignocellulose had the effect of increasing the live weight of birds (25, 35, 39). According to Sarikhan *et al*. (39) insoluble fibre may have a beneficial effect on the height of the villi in the digestive tract through a stimulation effect, and on the ratio of villi to crypts, leading to better absorption and retention of nutrients, which may ultimately lead to increased growth of birds and higher-yielding chicken broiler carcasses. However, some studies have shown that the use of compound feed supplemented with lignocellulose at lower levels of incorporation up to 2% did not in any way affect the productive performance of broiler chickens (5, 20, 35, 47). The use of a 1% insoluble fibre additive in feed for broiler chickens can increase liver weight (46, 48).

Leaving aside white striping, general meat colour is one of the most important factors influencing a consumer’s purchasing decisions. Even though variation in colour within a typical range is not a factor that can predict the quality and safety of the meat purchased, the consumer directly associates it with the subsequent quality of the food prepared. The inability of the potential consumer to detect differences in colour between the breast muscles from the experimental groups and the breast muscles from the control group completely rules out the possibility of a consumer’s visual preference being a reason for rejection of CFC-fed chicken meat.

White striping myopathy is currently one of the most important quality problems emerging in the poultry industry, and unfortunately its incidence is rapidly increasing in breast muscle (2, 9, 23, 34). Breast muscle with a WS defect is characterised by white parallel lines running in the same direction as the muscle fibres, and their number and thickness can vary from bird to bird. These white stripes have been reported to be composed of adipose tissue (8, 9, 38). Their presence is easily perceived and tends to deter consumers, as they give breast fillets a greasy, marbled and abnormal appearance (2, 9, 22). Unfortunately, the lack of publicly available literature sources makes it difficult to contextualise the results obtained in our studies and frame a discussion. The aetiology of WS has not yet been identified, but there are nevertheless several factors that may influence the incidence of this pectoral muscle myopathy: genotype (high breast yield > standard), sex (males > females), growth rate (fast > slow), slaughter weight (heavy > light) and diet (high-energy > low-energy) (10, 24, 34). It is the diet-related factor that could substantiate one potential mechanism for the effect of a CFC additive in feed on the reduction of WS incidence. The faster growth rate induced by using a high-calorie mix in the feeding of broiler chickens could increase the incidence of white stripes in broiler breast fillets (21). Referring to the results obtained by the team of Kuttapan *et al*. (21), inversely it can be assumed that feeding a low-energy mixture to broiler chickens reduces the occurrence of WS myopathy. By definition, a low-energy mixture is one in which the energy level has been reduced by using, for example, a dilution factor. Therefore, the crude fibre concentrate used to replace part of the feed most likely acted as a diluting element by distributing the energy level of the feed differently and transforming it into a mixture with a lower energy level, which could directly affect the occurrence of WS.

No significant differences were found between any group and any other for the rest of the results obtained from the analyses. These results are typical of the literature data relating to the body component percentages of the measured broiler organs and physicochemical parameters of the pectoral muscle.

Based on the obtained results, it was found that the addition of CFC in the feed mixture for broiler chickens increased the final body weight, carcass weight and the percentage of live weight which was leg muscle in both experimental groups in comparison to the control group. Crude fibre concentrate was also noted to raise the percentage of live weight which was liver in group A2 in comparison to group C and lower the percentage of breast muscle tissue affected by WS in both experimental groups in comparison to the control group. The feeding strategy had no adverse effect on the other quality parameters of breast muscle.

The results obtained from the analyses provide answers to many questions that arose before and during the experiment. However, they also bring many additional questions, such as how ARBOCEL crude fibre concentrate used in the diet of broiler chickens may affect the fatty acid profile, the content of individual amino acids and the content of consumer-important micro- and macroelements in the breast muscle analysed. In addition, it would be worthwhile to focus on fully understanding the possible mechanism of the effect of CFC-containing feed on reducing the incidence of thoracic muscle myopathy. Unfortunately, there is still a lack of specific studies in the available literature on the effect of the addition of CFCs on the quality of meat obtained from reared broiler chickens, which, on the one hand, thwarts discussion, but on the other hand, offers many opportunities for further scientific research.

## Conclusion

The addition of CFC to the complete feed mixture is recommended for better slaughter analysis results and better meat visual quality in the aspect of reduction in the number of muscles characterised by white striping. The results obtained from the analyses conducted in the experiment provide a broader perspective on the addition of crude fibre concentrate. Introducing CFC to feed carries several benefits for producers, *e.g*. increased final average live weight of birds, reduced incidence of welfare disorders such as footpad dermatitis, and better production indices such as the European Production Efficiency Factor and the European Broiler Index (44). The benefit associated with using the CFC additive in the feed mix may also influence the subsequent consumer decision to purchase chicken breast muscle, as it can be stated that “the consumer buys with his eyes”. Therefore, reduction of the incidence of WS myopathy makes the consumer more likely to choose breast muscle without visual manifestation of it when making his choice. However, to fully benefit from the potential of CFC in broiler feed, the producer should follow the manufacturer’s proportion recommendations.
